# The presence of glutathione S-transferase in recombinant S100A9 alters its effect on human sperm function

**DOI:** 10.7555/JBR.38.20240155

**Published:** 2025-02-08

**Authors:** Estefania Massa, Gastón Prez, Sergio Ghersevich

**Affiliations:** Area of Clinical Biochemistry, Facultad de Ciencias Bioquímicas y Farmacéuticas, Universidad Nacional de Rosario, Suipacha 531, (2000) Rosario, Santa Fe, Argentina

Dear Editor,

In a recent study, we isolated a protein from human oviductal secretion that could bind to spermatozoa^[1]^. This protein was identified through chromatography and tandem mass spectrometry as human S100A9 and was detected in human tubal epithelium and oviductal secretions.

S100A9 belongs to the S100 protein family^[2]^, which has been found in various body fluids and tissues, and plays a role in extracellular functions, such as the enhancement of neutrophil extravasation, induction of proinflammatory cytokine release, antimicrobial properties through divalent ion sequestration, and modulation of cellular proliferation, differentiation, and apoptosis, as well as acting as a chemotactic factor^[3–4]^. Because S100A9 is involved in various pathologies and the physiology of inflammation, research on S100A9 effects continues to grow rapidly. Recently, we have shown the presence of binding sites for S100A9 on human spermatozoa, and also found that S100A9 modulated certain sperm capacitation parameters *in vitro*, such as the induced acrosome reaction (AR)^[1]^. To continue our studies on sperm function parameters, the current study aimed to express and purify human recombinant S100A9 and to assess its effect on sperm capacitation parameters, specifically the AR.

Because S100A9 does not have glycosylated amino acid residues, the cloning and expression of human *S100A9* were carried out in an expression vector in bacteria. The human *S100A9* cDNA was inserted into the PGEX-2T plasmid (Cat. #V010921, NovoPro Bioscience Inc., Shanghai, China), alongside a built-in sequence of glutathione S-transferase (GST), and was cloned in *Escherichia coli*. The expressed GST-S100A9 was purified using glutathione-agarose (GSH-A; Cat. #16100, Pierce, Thermo Scientific, Waltham, MA, USA). Subsequently, the fusion protein was treated with agarose-thrombin beads (Cat. #RECOMT-1KT, Thrombin-A, Thrombin CleanCleave Kit, Sigma-Aldrich, St. Louis, MO, USA) to remove the GST moiety (pS100A9). The recombinant proteins were detected by Western blotting analysis using a rabbit anti-human S100A9 antibody (Cat. #sc-58706, Santa Cruz Biotechnology, Santa Cruz, CA, USA), which has demonstrated high specificity and sensitivity.

**Figure 1:**
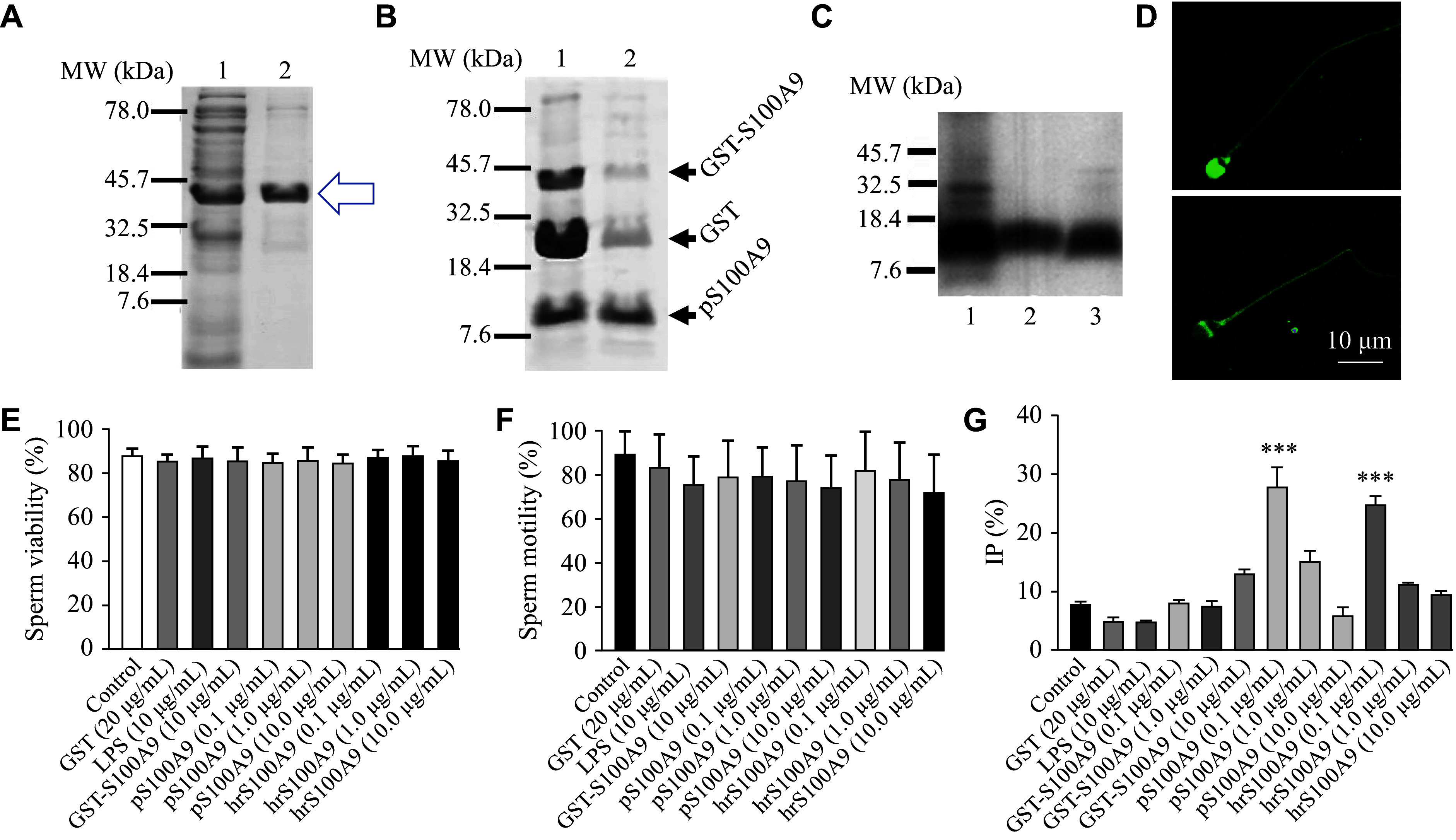
Expression and isolation of human recombinant S100A9 and the effect on the acrosome reaction. A: SDS-PAGE (12%; Coomassie blue staining) analysis. Lane 1: cell lysate obtained after isopropyl β-D-1-thiogalactopyranoside induction. Lane 2: eluate after glutathione-agarose (GSH-A) purification. The arrow indicates the band of fusion protein (GST-S100A9). Both lanes exhibit a band corresponding to GST-S100A9. B: SDS-PAGE (15%; Coomassie blue staining) analysis. Arrows (from upper to lower) indicate the GST-S100A9, GST (approximately 23 kDa), and purified S100A9 (pS100A9) bands (approximately 13 kDa), respectively. Lane 1: the purified fusion protein treated with Thrombin-A. Lane 2: the sample from lane 1 was further treated with GSH-A, which showed that the fusion protein and GST were almost completely removed by the treatment. C: Detection of the expressed S100A9 by Western blotting. Lane 1: commercial human recombinant S100A9 (hrS100A9; 0.5 μg). Lane 2: human oviductal fluid (10 μg total protein). Lane 3: pS100A9 (1 μg). D: Representative fluorescence images of acrosome staining. Top: spermatozoon with an intact acrosome. Bottom: spermatozoon with a reacted acrosome. E: Sperm viability under different treatments. F: Sperm motility under different treatments. G: Effect of different recombinant S100A9 on acrosome reaction (AR), with the percentages of induced population (IP) in different treatment groups. After a six-hour incubation under capacitating conditions, two aliquots of sperm suspension from each treatment or control were taken: one was supplemented with progesterone (induced AR), and the other was supplemented with only the culture medium (spontaneous AR). The IP (%) for each treatment was calculated as follows: induced AR (%) − spontaneous AR (%). Three different controls were performed: basal control with only culture medium (Control), bacterial lysate control (lipopolysaccharides [LPS]), and GST control (GST). Data are presented as mean ± standard deviation (*n* = 5 experiments in duplicate). Data were analyzed with ANOVA and the Kruskal-Wallis multiple comparison test. ^***^P < 0.001 *vs.* Control.

The results indicated that the "in batch" purification method allowed the recovery of approximately 10 mg/mL of fusion protein per milliliter of GSH-A (***Fig. 1A***). The fusion protein was treated with the enzyme thrombin to separate S100A9 from GST. The gel analysis showed that, despite the purification steps, the sample still contained small amounts of residual GST and GST fusion protein (GST-S100A9) that did not bind to GSH-A (***Fig. 1B***). It is suggested that the target protein purity is usually greater than 90% at this stage^[5]^. Western blotting analysis revealed a strong band for the pS100A9 protein, demonstrating a molecular weight similar to that of the control human recombinant S100A9 (hrS100A9; Cat. #ab95909, Abcam Inc., Cambridge, MA, USA) and the native S100A9 from human oviduct fluid (***Fig. 1C***).

Human spermatozoa were obtained from normozoospermic donors (*n* = 5) through masturbation after three to five days of sexual abstinence^[6]^. Motile sperm obtained by the swim-up method were incubated with increasing concentrations (0, 0.1, 1.0, and 10.0 µg/mL) of each of the following proteins: hrS100A9, pS100A9, or GST-S100A9 in Ham's F10 medium (Cat. #11550043, Gibco, Grand Island, NY, USA), supplemented with 0.5% BSA at 37 ℃ with 5% CO_2_ for six hours. GST protein (20 μg/mL) and the lysate of untransformed bacteria (10 µg/mL lipopolysaccharides [LPS]) were used as controls. Subsequently, aliquots of each sperm suspension were incubated either in the absence (basal or spontaneous AR) or in the presence (induced AR) of 20 µmol/L progesterone at 37 ℃ with 5% CO_2_ for 30 min. Each experiment was performed in duplicate. The AR was detected using fluorescein isothiocyanate-*Pisum sativum* agglutinin labeling (***Fig. 1D***)^[1]^. The results were presented as the percentage of induced population (IP), which was calculated as the difference between induced AR (%) and basal AR (%). Analysis of variance (ANOVA) and the Kruskal-Wallis multiple comparison test were used to compare the means of IP (%), viability, and motility. A *P*-value < 0.05 was considered statistically significant.

Sperm viability was assessed by mixing one drop of 0.5% w/v Eosin Y solution with one drop of the sperm suspension on a microscope slide and examining it under a microscope to distinguish live (unstained) from dead (stained) sperm. A total of 200 spermatozoa were counted, and the percentage of viable cells was reported. For motility assessment, a 10 µL drop of sperm suspension was placed in a Makler chamber (Sefi-Medical Instruments, Haifa, Israel) and observed under a microscope. At least 200 spermatozoa from multiple fields were assessed. The percentage of motile sperm with forward progression was used for the analysis. The results showed that incubation with either GST-S100A9, pS100A9, hrS100A9, GST, or LPS did not affect sperm viability or motility, which were consistently higher than 85% and 70%, respectively (***Fig. 1E*** and ***1F***). These data suggest that the treatments have no cytotoxic effects on human spermatozoa under the experimental conditions and concentrations tested.

Spontaneous AR reflects the spermatozoa that undergo AR without the presence of an inducer, such as progesterone. Neither GST-S100A9 nor pS100A9 affected the spontaneous AR of sperm, as their values were similar to those of the control cells, while GST and LPS also did not affect spontaneous or induced AR (data not shown). These results suggest that the proteins do not directly affect acrosome stability. Notably, pS100A9 had an effect on AR similar to hrS100A9, with the lowest concentration of these proteins significantly increasing the IP (***Fig. 1G***). The dose-response effect of hrS100A9 and pS100A9 on the induced AR exhibited an inverted U-shaped behavior, where the highest dose did not affect the parameter, which is consistent with the potential mechanisms discussed by Massa *et al*^[1]^. Briefly, there may be changes in receptor availability or the presence of S100A9 oligomerization, which may reduce ligand-receptor binding, and thus affect the AR. In contrast, the fusion protein, even at the maximum concentration used, did not affect the induced AR in sperm, with values similar to those in the controls.

Regarding the use of GST-tagged recombinant protein, different studies in other cell models have used GST-S100A9 to assess its effects on various cell functions^[7-8]^. The fact that GST-S100A9 did not affect the AR may be attributed to several factors. It should be noted that the commercial hrS100A9 used was a recombinant protein with a His-tag at the C-terminus, lacking the GST moiety. Additionally, GST is typically a homodimeric protein, and the oligomer formed by GST and the fused protein may influence the properties of the latter^[5]^, possibly impeding its action. It appears that, when the GST moiety was removed, pS100A9 was able to interact with human sperm cells, leading to an increase in the induced AR. Thus, both pS100A9 and hrS100A9 further enhanced the progesterone-induced AR. Progesterone has been shown to stimulate calcium influx, the phosphorylation of sperm proteins, and an increase in cAMP, ultimately activating the sperm AR. In our previous study, we found that S100A9 bound to the head, principal piece, and midpiece of human spermatozoa and stimulated sperm protein tyrosine phosphorylation^[1]^. These binding sites overlap, at least partially, with the reported localization of toll-like receptor 4 (TLR-4) and the receptor for advanced glycation end products (RAGE) in the spermatozoon. It can be hypothesized, therefore, that the interaction of the protein with these receptors may mediate the effects on AR. Studies in various cell models have reported that S100A9 interacts with TLR-4 and RAGE^[9]^. Both receptors have been detected in sperm cells, with TLR-4 specifically located in the acrosome, midpiece, and tail, whereas RAGE is detected in the head and equatorial segment as well as a small population within the tail^[10]^.

It should be noted that, in the current study, sperms were incubated with various treatments under capacitating conditions for six hours before evaluating the AR. This approach reflects the effects of these treatments on the sperm capacitation process, as evidenced by the ability to undergo AR. At the end of incubation, the AR-inducer progesterone was added in the presence of the different treatments for 30 min. In other words, the proteins under study were not simply evaluated as AR-inducing agents during a short incubation time of 30 min.

In conclusion, while some studies in other cell models have used GST-tagged proteins to assess their effects on cell functions, our findings indicate that only pS100A9 without the GST moiety can affect the induced AR, whereas the fusion protein does not. The presence of GST in the fusion protein may impede the activation mechanism by which S100A9 modulates the induced AR.

Yours sincerely,Estefania Massa, Gastón Prez, Sergio Ghersevich^✉^ Area of Clinical Biochemistry, Facultad de Ciencias Bioquímicas y Farmacéuticas, Universidad Nacional de Rosario, Rosario, Santa Fe 2000, Argentina.^✉^Corresponding author: Sergio Ghersevich. E-mail: sghersevich@gmail.com.
